# *S. haematobium* as a Common Cause of Genital Morbidity in Girls: A Cross-sectional Study of Children in South Africa

**DOI:** 10.1371/journal.pntd.0002104

**Published:** 2013-03-21

**Authors:** Ingrid Elise Amlie Hegertun, Kristin Marie Sulheim Gundersen, Elisabeth Kleppa, Siphosenkosi Gift Zulu, Svein Gunnar Gundersen, Myra Taylor, Jane D. Kvalsvig, Eyrun Floerecke Kjetland

**Affiliations:** 1 Centre for Imported and Tropical Diseases, Department of Infectious Diseases Ullevaal, Oslo University Hospital, Oslo, Norway; 2 University of Oslo, Oslo, Norway; 3 School of Public Health Medicine, Nelson R Mandela School of Medicine, University of KwaZulu-Natal, Durban, South Africa; 4 Research Department, Sorlandet Hospital HF, Kristiansand, Norway; 5 Institute for Development Studies, University of Agder, Kristiansand, Norway; 6 School of Biological and Conservation Sciences, University of KwaZulu-Natal, Durban, South Africa; Weill Cornell Medical College, United States of America

## Abstract

**Background:**

*Schistosoma (S.) haematobium* infection is a common cause of genital morbidity in adult women. Ova in the genital mucosal lining may cause lesions, bleeding, pain, discharge, and the damaged surfaces may pose a risk for HIV. In a heterogeneous schistosomiasis endemic area in South Africa, we sought to investigate if young girls had genital symptoms and if this was associated with urinary *S. haematobium*.

**Methodology:**

In a cross-sectional study of 18 randomly chosen primary schools, we included 1057 schoolgirls between the age of 10 and 12 years. We interviewed assenting girls, whose parents had consented to their participation and examined three urines from each of them for schistosome ova.

**Principal findings:**

One third of the girls reported to have a history of genital symptoms. Prior schistosomal infection was reported by 22% (226/1020), this was associated with current genital symptoms (p<0.001). In regression analysis the genital symptoms were significantly associated both with urinary schistosomiasis (p<0.001) and water contact (p<0.001).

**Conclusions:**

Even before sexually active age, a relatively large proportion of the participating girls had similar genital symptoms to those reported for adult genital schistosomiasis previously. Anti-schistosomal treatment should be considered at a young age in order to prevent chronic genital damage and secondary infections such as HIV, sexually transmitted diseases and other super-infections.

## Author Summary

Urogenital schistosomiasis (Bilharzia) is a common cause of gynecological disease in adult women. Reports to date indicate that genital lesions in adults become chronic and that the damages make women susceptible to HIV. This is the first study on urogenital schistosomiasis in pre-pubertal girls. We interviewed girls aged 10 to 12 years of age for urinary and gynecological symptoms. The research assistants did not know the schistosomiasis infection status in the school or the individuals. We collected three urines that were examined for schistosome eggs. We found that a significantly increased number of girls with urinary schistosomiasis have stinking, bloody discharge, ulcers, tumors and a burning sensation in their genitals. This indicates that gynecological damages due to schistosomiasis start before sexual activity, and before menstruation. By preventing urogenital schistosomiasis in girls we may have an innovative opportunity to reduce teenage HIV transmission and gynecological disease. This study presents a new aspect of a neglected disease affecting more than 100 million females, long overdue for mass intervention.

## Introduction

Urogenital schistosomiasis causes gynecological morbidity in adult women [1], [2]. *Schistosoma (S.) haematobium* is primarily known for its effect on the urinary tract, but in endemic areas schistosomiasis may be the most common cause of genital morbidity and mucosal lesions [3]. An estimated 390 million females are at risk of schistosomiasis infection [4], [5]. It is second only to malaria in terms of public health impact of the parasitic diseases, with more than 100 million females infected, 85% of them live in rural parts of Africa.

Previous studies on urogenital schistosomiasis have been conducted in adult women of childbearing age. *S. haematobium* ova when deposited in the female reproductive tract seem to be equally distributed in the different genital parts, but are most commonly identified in the cervix and the vagina [6], [7], [8], [9]. Both viable and dead ova may cause tissue reactions, morbidity and symptoms long after contact with infested waters [10], [11]. The disease may manifest itself in both the genital and urinary tract and may be found exclusively in the genitals [6], [12]. In young girls there have only been a few case reports, hypothesizing that the pre-pubertal predilection site is in the vulva [7], [8], [9], [13], [14]. This may partly be because gynecological inspections are not prioritized in rural areas, controversial in virgins, but also because the causal relationship between schistosomiasis and genital lesions in young females has not been explored on a large scale [14], [15].

It has been hypothesized that female genital schistosomiasis poses an increased risk to secondary infections such as human papillomavirus and other STDs. Most importantly, these women have been found to have significantly more HIV [16], [17], [18], [19]. In the wake of the HIV epidemic and a realistic prospect of successful anti-schistosomal mass-treatment programs this study sought to explore if girls before sexual debut had signs of genital disease [20]. In Ugu District, South Africa the study aimed to explore the association between gynecologic symptoms and urinary *S. haematobium* in young girls.

## Methods

### Study design and participants

We carried out a cross-sectional study in 18 primary schools, which were randomized for inclusion from 309 primary schools in the area. We invited all girls aged 10–12 years in the included schools. Girls who were absent on the days of the invitations were excluded, as were girls with serious illnesses, or if their guardians or they refused.

### Setting

**Figure 1:**
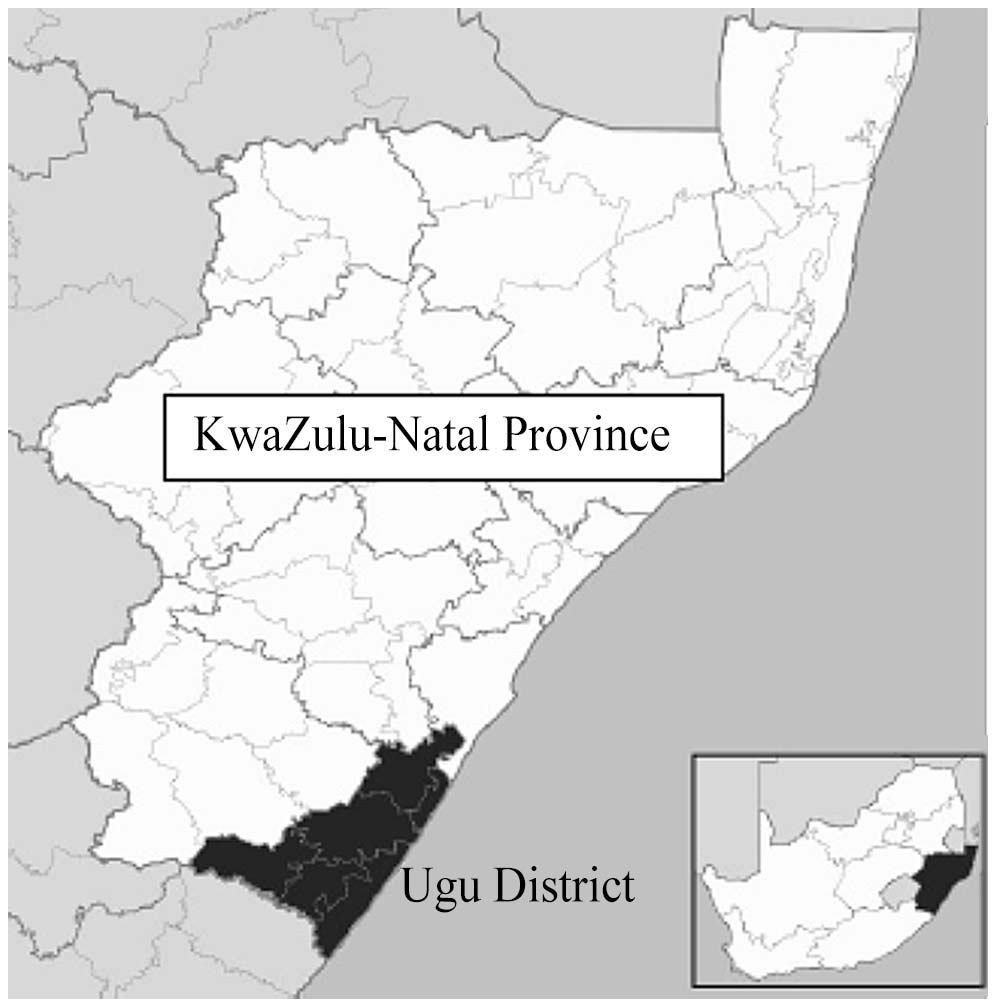
Map of Ugu district in South Africa. The coastal areas are inhabited by the more affluent and the schools here were excluded.

The schools were visited between September 2009 and November 2010 in the predominantly rural Ugu District, KwaZulu Natal, South Africa, an *S. haematobium* endemic area, which covers 5866 km^2^ (Figure 1). It has an estimated population of 700 000 almost exclusively isiZulu speaking people, 84% reside in the rural areas, 51% are below the age of 20 years and 55% are female [21].

### Ethical considerations

The study was approved by The Biomedical Research Ethics Administration, UKZN 2009, Ref BF029/07; by the Department of Health, Pietermaritzburg, 2009, Ref HRKM010 - 08; by the Norwegian ethics committee, Ref 469 - 07066a1.2007.535, 2007, and the Departments of Health (2008) and Education (2009) in Ugu District. The Helsinki Declaration was followed. All members of the group, including students and research assistants had passed exams in Good Clinical Practice and signed Declarations of Confidentiality. The interviewers did not know the study subjects beforehand. Prior to the study there were information meetings for the parents, principals, school governing bodies and/or teachers of each school. Informed consent was given by each girl, and the parents/guardians signed consent forms. Identifying information was stored separate from the interview information (in separate towns). All were informed about their right to withdraw and to abstain from answering questions without negative consequences. In order to protect girls from stigmatization the disease was discussed in general terms as urinary schistosomiasis. Treatment for schistosomiasis was offered to all, and all were informed about possible side effects. A private psychologist was hired by the project to take care of referred cases as felt necessary; for psychological, practical and legal issues. When other medical help was required, the girls were referred to a government clinical facility, or offered private care if government services were unavailable. For ethical and community liaison reasons the project staff was not involved in any physical or psychological examinations after referral.

### The interview

The interviews (30 minutes duration) were conducted face to face in isiZulu (the local language) by trained female fieldworkers. Questions were asked about recent (the last week) or previous urogenital symptoms of itch, burn, ulcers or tumors (swelling, lumps) in the genitals, malodorous discharge, color of discharge and feeling of a burning sensation in the genitals, as well as red urine, dysuria, urge and stress incontinence [1], [2]. They were also asked questions about confounders for bloody discharge (menstruation and red urine), malodorous discharge (sexual intercourse and sexual abuse) and for burning sensations in the genitals (sexual intercourse and dysuria). The girls in a pilot study (same age) had no concept of the local anatomy and we therefore decided to not include questions on exact localization of e.g. tumors. In case the child did not seem to understand, terms were explained and if she seemed too shy/uncomfortable to answer the interviewers were instructed to move to the next question. The discharge color was defined using a custom-made color chart. The study population was not familiar with the details of the questionnaire on beforehand. A water body was defined as a river, dam, lake, stream or pond. Each child was asked if she carried out any of seven specific water-related activities known in the study area (playing/swimming, washing/bathing, laundry, washing blankets, collecting water, fishing and crossing) [22]. Furthermore, there were validated demographic, social and psychological components in the questionnaire that will not be analyzed here.

### Parasitology

The researchers aimed to visit each school at least three times. We obtained urine samples from each girl on three consecutive days between 10 am and 2 pm. After gentle tilting we deposited 10 ml of urine into a container with 1 ml methylate-formalin solution and the same week we investigated the specimens by microscopy [23]. After centrifuging, we transferred all of the precipitate onto microscopy slides; the last amount was washed with water before transferred. If the mean number of eggs of the three specimens was higher than 50 per 10 ml urine the infection was classified as high-intensity [24]. One stool sample per child was collected for Kato Katz and analyzed for *Ascaris lumbricoides*, *Ancylostoma duodenale*, *Taenia solium*, *Trichuris trichiura* and *S. mansoni*. If there was at least one ovum in a specimen it was defined as positive.

### Data management and statistical analyses

Based on data from studies in adults we estimated the prevalence of genital schistosomiasis to be 30% and urinary schistosomiasis 40% [3]. We hypothesized that the expected prevalence of genital ulcers in the schistosomiasis exposed to be 9% and in the unexposed 4%. To detect a difference with a significance level of 5% and a power of 80%, the sample size would have to be 511 unexposed and 341 schistosomiasis exposed young women, in all 852 subjects. The information was recorded on paper; the personal information sheet was separated from the other information as soon as the record number had been secured. Data was entered into EpiData (interview) or Excel (urine) and subsequently exported into IBM SPSS version 19 (Chicago, Illinois, USA). Chi-square and odds ratios (OR) with 95% confidence interval (CI) were used to compare impacts of water contact or current urinary schistosomiasis infection on genital symptoms. In order to study the impact of other variables (for example menstruation or red urine), logistic regression analysis was applied with a 5% significance level; variables were included if the P-value in the crude association was less than 0.2 and if the Spearman rank correlation coefficient was below 0.7. When there were less than 10 cases, the variable was not included in regression analysis. The statistical analysis was computed using SPSS.

## Results

### Characteristics of the study group

Schools that were randomized for inclusion were visited in no particular order. We invited all pupils aged 10 to 12 years. All schools were visited several times in order to collect guardian consent forms and to find as many students as possible. The parents of 1241/1948 (64%) pupils in 18 schools provided consent. On the days we were in the schools we were able to include 1057 assenting girls. In the first 13 schools, where there was adequate time before exams, the consent forms were returned and signed by 92% (1109/1201) of the parents. The pupils were recruited from grades 1 to 7, median grade 5.

### Menstruation, HIV testing and sexual history

Answers were recorded as missing if the child chose not to answer or did not understand the topics. Seven percent of the girls (71/1019) had started menstruating. Only 5% (51/981) said they had been tested for HIV. Three out of 980 (0.3%) knew they had HIV, as many as 495 said they did not know and 77 girls did not reply to this question. Less than one percent (7/1017) reported to have had intra-vaginal sex. However, two percent (22/953) reported to have been sexually abused. They were referred to psychosocial follow-up. All in all 24 of 1019 young girls had experienced voluntary or involuntary vaginal sex.

### Parasitology

Out of the 1057 girls who were interviewed, 970 submitted at least one urine for examination, and out of these 791 submitted three urine samples. *S. haematobium* eggs were found in 32% (312/970) of the girls. High-intensity urinary infection was found in 28% (88/312) of these. In those who had ova in the urine, the mean intensity of infection was 52 eggs/10 ml urine (range 1–624/10 ml). Among the 658 girls with negative urine specimens, 79% (522) submitted three negative urine samples. There was neither any difference in urinary schistosomiasis infection intensity nor presence of symptoms between those who had submitted three urine samples versus one sample.

### Symptoms

Thirty five percent reported to have had genital symptoms (356/1018), and as many as 17% (172/1008) reported genital symptoms the last week. Eighteen girls reported having a genital tumor or an ulcer this last week. Table 1 shows the association between urinary schistosomiasis and symptoms in girls. Controlled for confounders in multivariate analyses the table shows that urinary schistosomiasis remained associated with bloody discharge, a burning sensation in the genitals, genital ulcers, tumors and incontinence. Having had vaginal sex was not significantly associated with any of the symptoms; however the variable ‘vaginal sex’ was forced into the multivariate analyses. It did not influence the association between the symptoms and urinary schistosomiasis or water contact. Likewise, having soil-transmitted helminths did not influence the associations (data not shown) and only one person had *S. mansoni*. The discharge color was white in 51% (84/164) of the cases; cream color in 35% (58/164) and yellow in 9% (14/164). The discharge had streaks/traces of red in 13% (11/87) of the cases, light red in 66% (57/87) of the cases and an even lighter shade of red (light pink) in 9% (8/87). Patients with symptoms were referred but not investigated by the project.

**Table 1:** Association between the urogenital symptoms in rural 10–12 year old girls and urinary schistosomiasis.

Symptoms and frequencies	In 298 urinary schistosomiasis positive^a^ (%)	In 628 urinary schistosomiasis negative (%)	OR (95% CI)^b^	P	Adj. OR (95%CI)^c^	P
Bloody discharge						
Never	247 (83)	608 (97)	1.0		1.0	
Sometimes	32 (11)	13 (2)	6.1(3.1–11.7)	<0.001	4.2 (2.1–8.5)	<0.001
Always	19 (6)	7 (1)	6.7 (2.8–16.1)	<0.001	3.3 (1.3–8.5)	0.01
Red urine^d^						
Never	207 (69)	568 (90)	1.0		1	
Sometimes	37 (12)	34 (5)	2.9 (1.8–4.9)	<0.001	2.4 (1.5–4.1)	0.001
This week	54 (18)	26 (4)	5.7 (3.5–9.3)	<0.001	4.3 (2.5–7.2)	<0.001
Malodorous discharge						
Never	254 (85)	565 (90)	1.0		1.0	
Sometimes	20 (7)	39 (6)	1.1 (0.7–2.0)	0.64	1.1 (0.6–2.0)	0.69**
Always	24 (8)	24 (4)	2.2 (1.2–4.0)	0.007	2.2 (1.2–4.0)	0.008
Genital itch						
Never	237 (80)	524 (83)	1.0		1.0	
Sometimes	39 (13)	62 (10)	1.4 (0.9–2.1)	0.13	1.4 (0.9–2.2)	0.12**
This week	22 (7)	42 (7)	1.2 (0.7–2.0)	0.59	1.2 (0.7–2.0)	0.57**
Burning sensation in the genitals						
Never	243 (82)	561 (89)	1.0		1.0	
Sometimes	34 (11)	43 (7)	1.8 (1.1–2.9)	0.01	1.6 (1.0–2.6)	0.05
This week	21 (7)	24 (4)	2.0 (1.1–3.7)	0.02	1.9 (1.0–3.5)	0.05
Dysuria						
Never	220 (74)	516 (82)	1.0		1.0	
Sometimes	55 (18)	75 (12)	1.7 (1.2–2.5)	0.005	1.6 (1.1–2.3)	0.02
This week	23 (8)	37 (6)	1.5 (0.9–2.5)	0.17	1.3 (0.7–2.2)	0.42**
Genital ulcer						
Never	266 (89)	598 (95)	1.0		1.0	
Sometimes	27 (9)	25 (4)	2.4 (1.4–4.3)	0.002	2.4 (1.4–4.3)	0.002
This week	5 (2)	5 (1)	2.2 (0.6–7.8)	0.20	2.2 (0.6–7.7)	0.21**
Genital tumor						
Never	283 (95)	613 (98)	1.0		1.0	
Sometimes	12 (4)	13 (2)	2.0 (0.9–4.4)	0.09	2.0 (0.9–4.4)	0.09
This week	3 (1)	2 (0)	3.2 (0.5–19.6)	0.19	3.3 (0.5–19.6)	0.19**
Urge incontinence						
Never	184 (62)	443 (71)	1.0		1.0	
Sometimes	77 (26)	109 (17)	1.7 (1.2–2.4)	0.002	1.7 (1.2–2.4)	0.002
This week	37 (12)	76 (12)	1.2 (0.8–1.8)	0.47	1.2 (0.8–1.8)	0.44**
Stress incontinence						
Never	212 (71)	530 (84)	1.0		1.0	
Sometimes	44 (15)	66 (11)	1.7 (1.1–2.5)	0.02	1.7 (1.1–2.5)	0.02
This week	42 (14)	32 (5)	3.3 (2.0–5.3)	<0.001	3.3 (2.0–5.3)	<0.001

Eight separate multivariate analyses.

Age was forced into each model and did not influence the results (data not shown).

a The presence of at least one schistosome ova in any of the urine examined specimens.

b Odds ratio (OR) with 95% confidence interval (CI).

c Adjusted odds ratio, different confounding variables were included in each multivariate analysis for the specific genital symptom.

d Red urine as seen by the child.

** If recalculated as ‘ever had the symptom’ it is significantly associated with urinary schistosomiasis.

### Nuances in urinary schistosomiasis negative and positive groups

**Figure 2:**
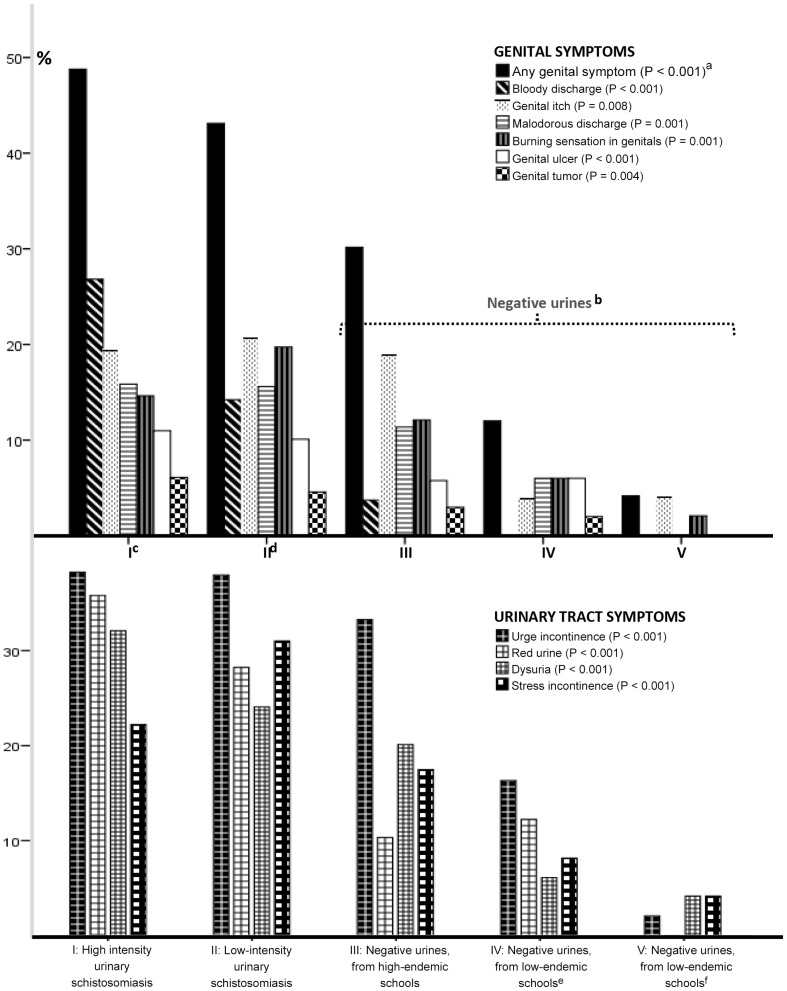
Genital and urinary symptoms in girls of two *S. haematobium* positive groups and three negative risk groups. ^a^Likelihood ratio. ^b^Three urines investigated for *S. haematobium* ova, all were negative. ^c^More than 50 *S. haematobium* ova per 10 ml urine. ^d^1–49 ova per 10 ml urine. ^e^These girls have water body contact (e.g. river, dam or lake). ^f^These girls deny water body contact.

In order to explore the urinary negative girls in more detail they were first divided into two groups (Figure 2), those in high-endemic school versus those in low endemic. The pupils from the low-endemic schools were further split into two, those who admitted water body contact and those who did not. Figure 2 shows that symptoms are significantly more common in those that have a high intensity of infection. The figure also highlights that low-endemic schools (the ‘most negative’ in the district) have a low prevalence of genital symptoms. Amongst the girls with high-intensity schistosomiasis almost 50% had genital symptoms, compared to less than 5% in the negative girls who lived in non-endemic areas and had no water contact. These girls denied having genital tumors, ulcers, bloody or smelly discharge. Bloody discharge was found in the high-endemic schools only, notably also in those individuals of these schools who were negative for schistosomiasis in three urines (Figure 1, category III). Urge incontinence and genital itch were both relatively constant in the high-endemic schools, but significantly higher than in the low-endemic schools.

### History of water contact

We found a significant association between water contact and all the listed symptoms (data not shown). However, among the 364 who denied water contact, 21% (76) had urinary schistosomiasis. Sixty three percent of the girls reported water contact (667/1057), among these 606 submitted urines and 39% (236/606) had *S. haematobium* ova in urine. Among the girls with three negative urines, 54% (281/522) reported water contact. These reported significantly more genital symptoms the last week than their peers without water contact (p = 0.001).

### Prior urinary infection with *S. haematobium* and symptoms

Twenty two percent of the young girls (226/1020) reported having had urinary schistosomiasis previously. This was significantly associated with current genital symptoms such as bloody discharge (Chi square, p<0.001), malodorous discharge (p<0.001), genital itch (p<0.017) and genital ulcer (p = 0.001). Furthermore 29% (251/853) knew of a family member who had had urinary schistosomiasis or red urine and these factors too were associated with all the queried symptoms (p≤0.002), except genital tumor (p = 0.08).

Twelve percent (129/1057) reported that they had been treated for schistosomiasis previously. Girls who said that they had not been treated had significantly more urinary schistosomiasis than those who had been treated (p<0.001), but not more symptoms (sample size small and p-values range from p = 0.43 to p = 1.0).

## Discussion

In an *S. haematobium* endemic area girls aged 10 to 12 years with schistosomiasis had significantly more often unpleasant symptoms such as genital ulcers, bloody discharge, malodorous white to yellow cultured discharge, genital itch or tumors than those without this infection. Even before sexual debut and independent of menstruation more than 40% of girls with *S. haematobium* ova in urine reported having had gynecological symptoms previously, one third reported having it the last week or ‘always’. Girls living in endemic areas without urinary schistosomiasis also had significantly more genital symptoms than their peers in low-endemic schools. This study shows that urinary schistosomiasis, water contact, history of red urine and family history of schistosomiasis (‘Isichenene’ in Zulu) are also associated with the full range of symptoms. As shown in adults previously, the history of water contact was an excellent predictor for genital symptoms also in girls [3], [7], [8], [9], [25]. The girls who had been treated for schistosomiasis previously had the same symptoms as those who denied having received treatment.

In adults the grainy sandy patches have been found to be diagnostic of *S. haematobium* infection and are significantly associated with discharge [1], [2], [3]. However, the findings in this young population could not be corroborated by a clinical examination. These results are therefore circumstantial, since the gynecological symptoms are not specific for genital schistosomiasis. Without the physical examination and intravaginal tests we cannot confirm schistosomiasis as the etiological factor. Furthermore, vaginal discharge, ulcers and genital itch may have other causes that were not controlled for in this study, such as the sexually transmitted diseases, atopic, irrigative dermatitis or other dermatoses like psoriasis or lichen sclerosis, lice, scabies, or non-specific etiology [26], [27]. Furthermore, one cannot preclude that the current symptoms, although caused by infection with *S. haematobium* in the lower genital tract may make the genital mucosa more susceptible to super-infections by other agents such as bacterial infections, which in turn may cause the reported symptoms. Likewise, the association between water contact and symptoms may be influenced by social and other practices. Poor perinea hygiene may be more common in a group that has limited access to water; and cultural cleansing rituals may also be hypothetical reasons for the association between water contact and symptoms [28].

In this study three urines were collected and the presence of schistosome ova defined the urinary schistosomiasis positive group. However, this study confirms that even urinary negative cases in endemic areas have gynecological morbidity. Hence the prevalence is most likely higher in this population. A more sensitive diagnostic method, such as antigen detection or PCR, would likely have made the reported finding more apparent, though this was not possible in our study [29].

Some girls denied having had water contact, but were found to still have *S. haematobium* ova in urine. Girls may be shy, worried about repercussions or not be able to differentiate between urinary and genital symptoms. Some girls were ignorant of some phenomena in the questionnaire such as menstruation or discharge. The interviewers – all female – were trained to explain the differences, however information sessions using dolls followed by more thorough questioning of genital symptoms could perhaps have produced more reliable answers and less under-reporting. This was not done in the present study. Further, the most reliable method to determine water contact is by direct observation, although many schistosomiasis studies have used self-reported water body data [30], [31].

It is well documented that many adult women may have genital schistosomiasis even without having detectable schistosome ova in the urine [3], [12], [32]. Urine investigations may therefore be of limited use in the diagnosis of genital schistosomiasis. Studies have shown that female genital schistosomiasis may cause pathologic blood vessel morphology and fragile blood vessels that may lead to mucosal bleeding [3], [33], [34]. Bloody discharge may be a result of this. Inter-menstrual bleeding, post-coital bleeding, malodorous and abnormally colored discharge and genital itch have been found to be associated with *S. haematobium* ova in the genitals of adults, even after correcting for sexually transmitted diseases [1], [19], [25], [34].

The girls in this study report the same symptoms as adult women in previous studies [1], [2]. One may fear that young children's genital mucosa are already imbued with calcified *S. haematobium* ova [16], [25], [35]. Childhood water contact may start very early and these girls may have had *S. haematobium* infection for several years [36]. One study found that such lesions were refractory to treatment in adults, whereas treatment received before the age of 20 years seemed to offer some protection against genital mucosal pathology [25]. Even so, the morbidity prevalence levels were unacceptably high even in those who had received treatment once in childhood and treatment may have to be given in infanthood in order to prevent genital damage [36]. Furthermore, siblings and people sharing the same water bodies should be given simultaneous treatment in order to reduce re-infection rate and intensity; treatment should be given in low-transmission seasons, and the effect should be secured by several rounds [37].

At the present time there are no suitable tools for the diagnosis of genital schistosomiasis in girls. Abnormal malodorous or bloody genital discharge are mucosal symptoms [2]. In our young study population gynecological investigations were not possible for cultural and technical reasons [3]. Further studies are needed to triangulate the analyses of (1) symptoms and (2) water contact/family history with (3) the objective mucosal findings in genital schistosomiasis.

For the rural clinician history taking and urine analyses are simpler sources of information than gynecological examinations, and especially so in virgins. The findings in this study suggest that young girls in *S. haematobium* endemic areas have gynecological symptoms as a result of schistosoma infection. Mucosal damages may be present as these young girls enter into their first sexual relationships, making them particularly susceptible to HIV or human papillomavirus infection [16], [17], [36]. Further studies are needed to explore the effects of treatment on the prolific symptomatic manifestations and on decreasing the susceptibility to super-infections before sexual debut.

## Supporting Information

Checklist S1 Enclosed is a Strobe checklist for cross-sectional studies.
(DOC)
